# Motivators of Inappropriate Ovarian Cancer Screening: A Survey of Women and Their Clinicians

**DOI:** 10.1093/jncics/pkaa110

**Published:** 2020-12-08

**Authors:** Courtney Macdonald, Danielle Mazza, Martha Hickey, Morgan Hunter, Louise A Keogh, kConFab Investigators, Sandra C Jones, Christobel Saunders, Stephanie Nesci, Roger L Milne, Sue-Anne McLachlan, John L Hopper, Michael L Friedlander, Jon Emery, Kelly-Anne Phillips

**Affiliations:** Department of Medical Oncology, Peter MacCallum Cancer Centre, Melbourne, VIC, Australia; Sir Peter MacCallum Department of Oncology, University of Melbourne, Melbourne, Australia; Department of General Practice, Monash University, Melbourne, Australia; Department of Obstetrics and Gynaecology, University of Melbourne and the Royal Women’s Hospital, Melbourne, Australia; Centre for Biostatistics and Clinical Trials, Peter MacCallum Cancer Centre, Melbourne, Australia; Centre for Health Equity, Melbourne School of Population and Global Health, University of Melbourne, Melbourne, Australia; Sir Peter MacCallum Department of Oncology, University of Melbourne, Melbourne, Australia; The Research Department, Peter MacCallum Cancer Centre, Melbourne, Australia; ACU Engagement, Australian Catholic University, Melbourne, Australia; University of Western Australia, Crawley, WA, Australia; Department of Medical Oncology, Peter MacCallum Cancer Centre, Melbourne, VIC, Australia; Centre for Epidemiology and Biostatistics, Melbourne School of Population and Global Health, University of Melbourne, Melbourne, Australia; Cancer Epidemiology Division, Cancer Council Victoria, Melbourne, Australia; Precision Medicine, School of Clinical Sciences at Monash Health, Monash University, Clayton, Melbourne, Australia; Department of Medicine, St Vincent’s Hospital, University of Melbourne, Melbourne, Australia; Department of Medical Oncology, St Vincent’s Hospital, Fitzroy, Melbourne, Australia; Centre for Epidemiology and Biostatistics, Melbourne School of Population and Global Health, University of Melbourne, Melbourne, Australia; Prince of Wales Clinical School University of New South Wales, Sydney, Australia; Department of Medical Oncology, Prince of Wales Hospital, Randwick, NSW, Australia; Department of General Practice and Centre for Cancer Research, University of Melbourne, Victorian Comprehensive Cancer Centre, Melbourne, Australia; School of Primary, Aboriginal and Rural Health Care, University of Western Australia, Perth, Australia; Department of Medical Oncology, Peter MacCallum Cancer Centre, Melbourne, VIC, Australia; Sir Peter MacCallum Department of Oncology, University of Melbourne, Melbourne, Australia; Centre for Epidemiology and Biostatistics, Melbourne School of Population and Global Health, University of Melbourne, Melbourne, Australia

## Abstract

**Background:**

This study examined why women and doctors screen for ovarian cancer (OC) contrary to guidelines.

**Methods:**

Surveys, based on the Theoretical Domains Framework, were sent to women in the Kathleen Cuningham Foundation Consortium for Research into Familial Breast Cancer and family physicians and gynecologists who organized their screening.

**Results:**

Of 1264 Kathleen Cuningham Foundation Consortium for Research into Familial Breast Cancer women, 832 (65.8%) responded. In the past 2 years, 126 (15.1%) had screened. Most of these (n = 101, 80.2%) would continue even if their doctor told them it is ineffective. For women, key OC screening motivators operated in the domains of social role and goals (staying healthy for family, 93.9%), emotion and reinforcement (peace of mind, 93.1%), and beliefs about capabilities (tests are easy to have, 91.9%). Of 531 clinicians 252 (47.5%) responded; a minority (family physicians 45.8%, gynecologists 16.7%) thought OC screening was useful. For gynecologists, the main motivators of OC screening operated in the domains of environmental context (lack of other screening options, 27.6%), and emotion (patient peace of mind, 17.2%; difficulty discontinuing screening, 13.8%). For family physicians,, the strongest motivators were in the domains of social influence (women ask for these tests, 20.7%), goals (a chance these tests will detect cancer early, 16.4%), emotion (patient peace of mind, 13.8%), and environmental context (no other OC screening options, 11.2%).

**Conclusion:**

Reasons for OC screening are mostly patient driven. Clinician knowledge and practice are discordant. Motivators of OC screening encompass several domains, which could be targeted in interventions to reduce inappropriate OC screening.

Evidence does not support ovarian cancer (OC) screening in population-risk women. Randomized controlled trials have failed to demonstrate an improvement in survival with annual screening using transvaginal ultrasound (ultrasound) and cancer antigen 125 (CA125) in women at population risk of OC (1,2). In 2013, a meta-analysis (3) showed that screening did not improve survival and led to false-positive results requiring further investigation. Ultrasound resulted in a mean of 38 surgeries per OC detected, and 6% of women experienced surgical complications. Women with false-positive results experienced increased cancer-specific distress. These results are consistent with the most recent US Preventative Services Task Force statement, which recommends against screening for OC in average-risk women (4).

Despite the absence of survival benefit, women continue to undergo OC screening. Data from Norway and the United States suggest 21% to 58% of clinicians recommend screening to average-risk women (5-8). Previous research has demonstrated that clinicians who perform OC screening are driven by patient expectations, fear of litigation, and belief that OC screening reduces mortality (7). Women commonly overestimate their personal risk of OC (9), and most believe that screening improves survival (10).

*BRCA1* and *BRCA2* mutation carriers are considered high-risk for OC, with average lifetime risks of 44% and 17%, respectively (11). Bilateral salpingo-oophorectomy is associated with improved survival and is recommended as optimal risk reduction for this group (12-15). A prospective, nonrandomized trial in women at elevated risk suggested that use of an algorithm based on absolute level and rate of increase in CA125 had higher sensitivity for detection of early-stage OC than 6 monthly CA125 with a fixed cut point (16). Confirmatory studies are needed, and there is no evidence that OC screening in high-risk women improves survival.

International guidelines do not recommend OC screening for average and high-risk women. US National Comprehensive Cancer Network guidelines state that ultrasound combined with CA125 testing in *BRCA1* and *BRCA2* mutation carriers who do not undergo salpingo-oophorectomy can be considered at the clinician’s discretion from age 30-35 years; however, the benefit is uncertain (17). The UK guidelines recommend against OC screening outside a clinical trial (18,19). Australian guidelines state that there is no evidence to support OC screening in any population (20).

This study aimed to determine the prevalence of OC screening by Australian women enrolled in a familial breast cancer cohort, including women at average and elevated risk of OC. It examined knowledge and motivators of OC screening for women and the clinicians who organized their screening.

## Methods

### Setting

Participants were women (and their clinicians) from multiple-case breast cancer families who were recruited to the Kathleen Cuningham Foundation Consortium for Research into Familial Breast Cancer (kConFab) cohort between 1997 and 2008 (21,22). The proband was recruited after consultation in 1 of 15 Australian genetics clinics. Other family members could enroll in the cohort study without attending a genetics clinic. Women ranged from average to high risk of OC based on their family history and germline mutation status. Women are mailed follow-up questionnaires every 3 years (23) to update demographics (educational level, parity, and marital status), doctors involved in their care, cancer family history, cancer screening undertaken, risk reduction interventions, and cancer diagnoses (the latter 2 confirmed from pathology and surgical records). Clinician characteristics were obtained from the Australian Health Practitioner Regulation Agency website (24). All participants provided written informed consent, and kConFab and this survey study have human research ethics committee approval.

### Surveys of kConFab Women and Clinicians

Understanding the factors that influence use of OC screening for both clinicians and patients is the first step in implementing practice change to reduce OC screening. The Theoretical Domains Framework (TDF) (25,26) was developed to identify the cognitive, affective, social, and environmental influences on health professionals and patient behavior related to implementation of evidence-based recommendations. It consists of 84 theoretical constructs grouped into 14 domains, and maps directly to the capability, opportunity, motivation and behavior (COM-B) model (27), to suggest intervention functions and policy categories to guide potential pathways to behavioral change.

A 33-item survey (Supplementary Materials, available online) was developed based on a literature review and semistructured interviews with 62 kConFab women from different geographical locations, socioeconomic status, and ethnicities to identify possible motivators for OC screening. Survey questions based on the TDF (Table 1) (25) were developed by the research team, which included experts in health sociology, qualitative research, gynecology, and primary care. The survey was piloted for usability with 9 kConFab women in face-to-face cognitive interviews and further refined. kConFab women were eligible for the survey if they were aged between 25 and 70 years, resided in Australia, had no previous bilateral oophorectomy or breast cancer and no invasive cancer in the past 6 years, and had not participated in the pilot. The survey was sent to kConFab women, and nonresponders were followed up 3 times.

**Table 1.** Relationship of TDF domain, COM-B system source of behavior, and intervention function^a^

TDF domain	Domain description	Statement		COM-B source of behavior	Intervention function
		kConFab women	Clinicians		
Social/professional role and identity	A coherent set of behaviors and displayed personal qualities of an individual in a social or work setting	These tests might improve the chance I will stay healthy for my family.	I would not want to conflict with the provided by another clinician.	Reflective motivation	Education Persuasion Incentivization Coercion
Goals	Mental representations of outcomes or end states that an individual wants to achieve	These tests might improve the chance I will stay healthy for my family.	There is a chance these tests will detect cancer early and lead to more successful patient outcomes.		
Beliefs about capabilities	Acceptance of the truth, reality, or validity about an ability, talent, or facility that a person can put to constructive use	These tests are easy enough to have.	Sometimes it is too hard to talk women out of it.		
Optimism	The confidence that things will happen for the best or that desired goals will be attained	I believe these tests might pick up ovarian cancer early.	I am optimistic that these tests will detect ovarian cancer at an early and potentially curable stage.		
Beliefs about consequences	Acceptance of the truth, reality, or validity about outcomes of a behavior in a given situation	I believe these tests might pick up ovarian cancer early.	It is better than doing nothing at all. I am concerned if my patient develops ovarian cancer she may take legal action.		
Intentions	A conscious decision to perform a behavior or a resolve to act in a certain way	Not applicable.	Not applicable.		
Reinforcement	Increasing the probability of a response by arranging a dependent relationship, or contingency, between the response and a given stimulus	Normal test results provide reassurance and peace of mind that I do not have ovarian cancer.	There are no adverse consequences for me when ordering these tests. I have no way of knowing if my approach to ovarian cancer screening is similar to other clinicians.	Automatic motivation	Persuasion Incentivization Coercion Environmental restructuring Modeling Enablement
Emotion	A complex reaction pattern, involving experiential, behavioral, and physiological elements, by which the individual attempts to deal with a personally significant matter or event	Normal test results provide reassurance and peace of mind that I do not have ovarian cancer.	I order these tests for patients’ peace of mind. It is hard to discontinue these tests in asymptomatic women who have been having ovarian cancer screening for several years. I am worried I might miss an ovarian cancer diagnosis.		
Memory, attention, and decision processes	The ability to retain information, focus selectively on aspects of the environment, and choose between 2 or more alternatives	There are currently no other screening options available and it is better than doing nothing. The other option is to have my ovaries removed, and I don’t want that at the moment.	Not applicable.	Psychological capability	Education Training Enablement
Knowledge	An awareness of the existence of something	There is no reliable way to detect ovarian cancer at an early and potentially curable stage. Screening for ovarian cancer can lead to unnecessary tests and surgery.	There is no reliable way to detect ovarian cancer at an early and potentially curable stage in asymptomatic women. CA125 blood tests and ovarian ultrasound scans can lead to unnecessary tests and surgery in asymptomatic women.		
Behavioral regulation	Anything aimed at managing or changing objectively observed or measured actions	Not applicable.	There are no adverse consequences for me when ordering these tests. I have no way of knowing if my approach to ovarian cancer screening is similar to other clinicians.		
Environmental context and resources	Any circumstance of a person’s situation or environment that discourages or encourages the development of skills and abilities, independence, social competence, and adaptive behavior	There are currently no other screening options available, and it is better than doing nothing. It is affordable. Healthcare professionals change their mind all the time about the best tests/guidelines. I don’t trust my health professional’s . I have previously had ovarian cancer symptoms.	There are currently no other options available for ovarian cancer screening. A CA125 blood test is a simple test. An ovarian ultrasound is a simple test.	Physical opportunity	Restriction Environmental restructuring Enablement
Social influences	Those interpersonal processes that can cause individuals to change their thoughts, feelings, or behaviors	My family/friends encourage me to have these tests. My family/friends ovarian cancer was detected through screening.	Women ask for these tests.	Social opportunity	Restriction Environmental restructuring Enablement
Skills	An ability or proficiency acquired through practice	Not applicable.	I am confident talking about ovarian cancer screening with my patients.	Physical capability	Training Enablement

a COM-B system= capability, opportunity, and motivation behavior system; TDF = Theoretical Domains Framework.

The 28-item survey (Supplementary Materials, available online) was developed by the research team, following a literature review and using the TDF (Table 1). Eligible clinicians were family physicians (FPs) or gynecologists identified by kConFab women as having ordered their screening tests and who were currently practicing in Australia with valid address. Nonresponders to the mailed questionnaire were followed up 3 times.

### Statistical Analysis

The survey responses for both clinicians and kConFab women were analyzed using descriptive statistics in R version 3.6.1 [R Core Team (2015) (28)]. Missing data were not imputed. For all numeric data, the mean (standard deviation), median (range), and interquartile range are provided. For categorical data, the count, percentages, and 95% confidence intervals are provided. The *t* tests and exact χ^2^ tests were calculated to examine whether there was evidence of a difference between responders and nonresponders for continuous and categorical variables, respectively. Tests were 2-tailed, and *P* values less than .05 were considered statistically significant.

## Results

### kConFab Women Survey

Of 4982 women unaffected with breast cancer when enrolled in kConFab between 1997 and 2008, 1097 declined follow-up. Of the remaining 3885 women, 332 died during follow-up, and 2289 were excluded (previous breast cancer [n = 1045], younger than age 25 years or older than age 70 years [n = 499], not residing in Australia or invalid address [n = 294], participation in pilot [n = 9], invasive cancer in the past 6 years [n = 61], or bilateral oophorectomy [n = 381]). Of 1264 eligible women, 832 (65.8%) responded. Table 2 describes the characteristics of responders and nonresponders. Responders were statistically significantly more likely to have a tertiary education compared with nonresponders (48.1% vs 34.5%; *P* < .001). There were no other statistically significant differences between respondents and nonrespondents. Only 34 (4.1%) of respondents were *BRCA1* or *BRCA2* mutation carriers. A quarter of women (n = 210) perceived their OC risk as high (5.4%) or moderately increased (19.8%). Of these 210, 51 had a *BRCA1* or *BRCA2* mutation and/or a first-degree relative with OC.

**Table 2.** Characteristics of responders and nonresponders (60 responders, 82 nonresponders)

Characteristics	kConFab women			Family physicians		Gynecologists	
	Responders No. (%)	Non-responders No. (%)	*P*	Responders No. (%)	Non-responders No. (%)	Responders No. (%)	Non-responders No. (%)
Age, years							
18-29	2 (0.2)	1 (0.2)	.05^a^	n/a	n/a	n/a	n/a
30-39	112 (13.5)	47 (10.9)		n/a	n/a	n/a	n/a
40-49	243 (29.2)	161 (37.3)		n/a	n/a	n/a	n/a
50-59	253 (30.4)	125 (28.9)		n/a	n/a	n/a	n/a
≥ 60	222 (26.7)	98 (22.7)		n/a	n/a	n/a	n/a
Year of first medical registration^c^							
1960-1969	n/a	n/a	n/a	2 (1.0)	2 (1.0)	3 (5.1)	4 (5.0)
1970-1979	n/a	n/a		30 (15.6)	33 (17.0)	7 (11.9)	13 (16.3)
1980-1989	n/a	n/a		66 (34.4)	53 (27.3)	22 (37.3)	22 (27.5)
1990-1999	n/a	n/a		49 (25.5)	50 (25.8)	14 (23.7)	19 (23.7)
≥ 2000	n/a	n/a		45 (23.4)	56 (28.9)	13 (22.0)	22 (27.5)
Missing	n/a	n/a		0	3	1	2
Sex^c^							
Female	832 (100)	432 (100)	n/a	134 (69.8)	127 (64.5)	22 (36.7)	23 (28.0)
Male	0	0		58 (30.2)	70 (35.5)	38 (63.3)	59 (72.0)
*BRCA* mutation status							
*BRCA1* or *BRCA2*	34 (4.1)	27 (6.2)	.10^a^	n/a	n/a	n/a	n/a
No mutation	798 (95.9)	405 (93.8)		n/a	n/a	n/a	n/a
1^st^ degree relative affected with OC							
Yes	52 (6.2)	26 (6.0)	.90^b^	n/a	n/a	n/a	n/a
No	780 (93.8)	406 (94.0)		n/a	n/a	n/a	n/a
Marital Status							
Married/Living as married	628 (75.5)	313 (72.9)	.35^a^	n/a	n/a	n/a	n/a
Other	204 (24.5)	116 (27.1)		n/a	n/a	n/a	n/a
Missing	1	3		n/a	n/a	n/a	n/a
Parity							
Nulliparous	154(18.5)	69 (16.0)	.45^a^	n/a	n/a	n/a	n/a
1	212 (25.5)	107 (24.8)		n/a	n/a	n/a	n/a
≥ 2	466 (56.0)	256 (59.2)		n/a	n/a	n/a	n/a
Educational level							
Less than tertiary	431 (51.9)	285 (65.5)	<.01^a^	n/a	n/a	n/a	n/a
Tertiary	400 (48.1)	147 (34.5)		n/a	n/a	n/a	n/a
Missing	1	0		n/a	n/a	n/a	n/a
Perceived risk^d^							
High	45 (5.4)	n/a	n/a	n/a	n/a	n/a	n/a
Moderately increased	165 (19.8)	n/a		n/a	n/a	n/a	n/a
Average	520 (62.5)	n/a		n/a	n/a	n/a	n/a
Low	40 (4.8)	n/a		n/a	n/a	n/a	n/a
Don’t know	62 (7.5)	n/a		n/a	n/a	n/a	n/a

a Two-sided χ^2^ test. CI = confidence interval; kConFab = Kathleen Cuningham Foundation Consortium for Research into Familial Breast Cancer; N/A = not asked/not applicable.

b 2-sided Fisher exact test.

c Year of medical registration and sex were not statistically significantly different between clinician responders and nonresponders.

d High: more than 3 times that of most other women. Moderately increased: about 2 or 3 times that of most other women. Average: about the same as most other women. Low: lower than most other women.

**Figure 1.**
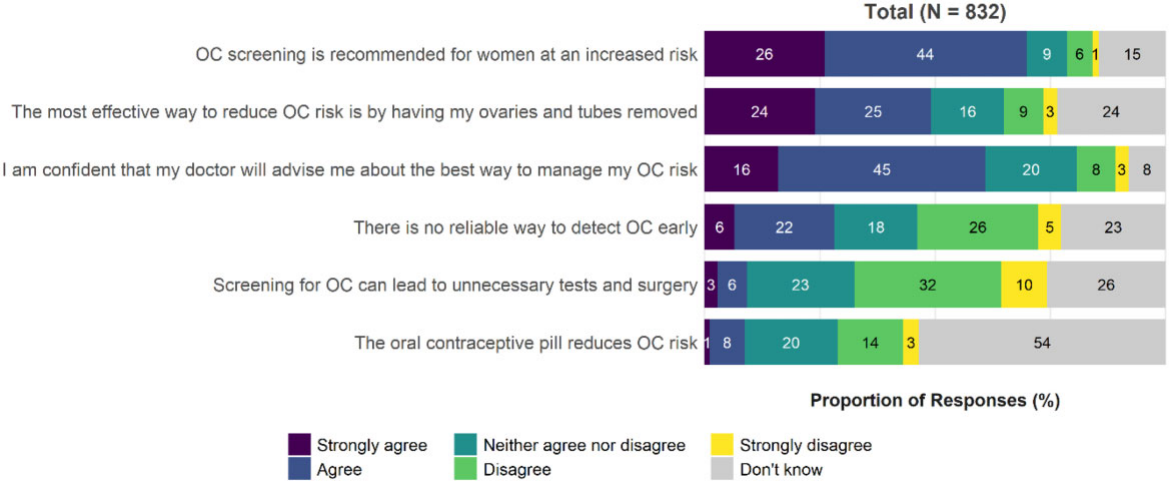
kConFab women: knowledge about ovarian cancer (OC) screening and risk reduction. kConFab = Kathleen Cuningham Foundation Consortium for Research into Familial Breast Cancer.

The majority of kConFab women (74.0%) reported that the use of CA125 and ultrasound in combination was either “highly likely” or “likely” to detect early-stage OC (46.1% highly likely, 27.9% likely). All respondents reported their level of agreement with statements about OC screening and risk reduction (Figure 1;Supplementary Table 1, available online). Many (41.9%) disagreed with the statement that OC screening can lead to unnecessary tests and surgery. Most (69.9%) agreed with the statement that OC screening is recommended for women at increased risk (26.0% strongly agreed, 43.9% agreed).

A minority of women (n = 126, 15.1%) had undergone screening in the past 2 years, with 103 (12.4%) having had an ultrasound, most (n = 85, 82.5%) arranged by FPs. More than one-third of *BRCA1* or *BRCA2* mutation carriers (n = 13, 38.2%) had undergone an ultrasound for screening compared with 11.3% (n = 90) of those who did not have a known *BRCA1* or *BRCA2* mutation. A minority of women (n = 53, 6.4%) had CA125 testing for OC screening in the past 2 years, including 4 (11.8%) *BRCA1* and *BRCA2* mutation carriers. The majority were arranged by FPs (n = 44, 83.0%) or gynecologists (n = 6, 11.3%).

Of the 45 (5.4%) women who perceived their OC risk as high, 18 (40.0%) had an ultrasound, 6 (13.3%) had CA125, and 4 (8.8%) had both. Almost half (n = 20, 44.4%) had undergone some form of OC screening compared with 24.8% of those who perceived their risk as moderate and 10.8% of those who thought their risk was average or below.

The majority (n = 101, 80.2%) of women who had undergone OC screening in the past 2 years responded that they would continue screening even if their doctor advised that there was no test that detects OC when it is early and potentially curable.

**Figure 2.**
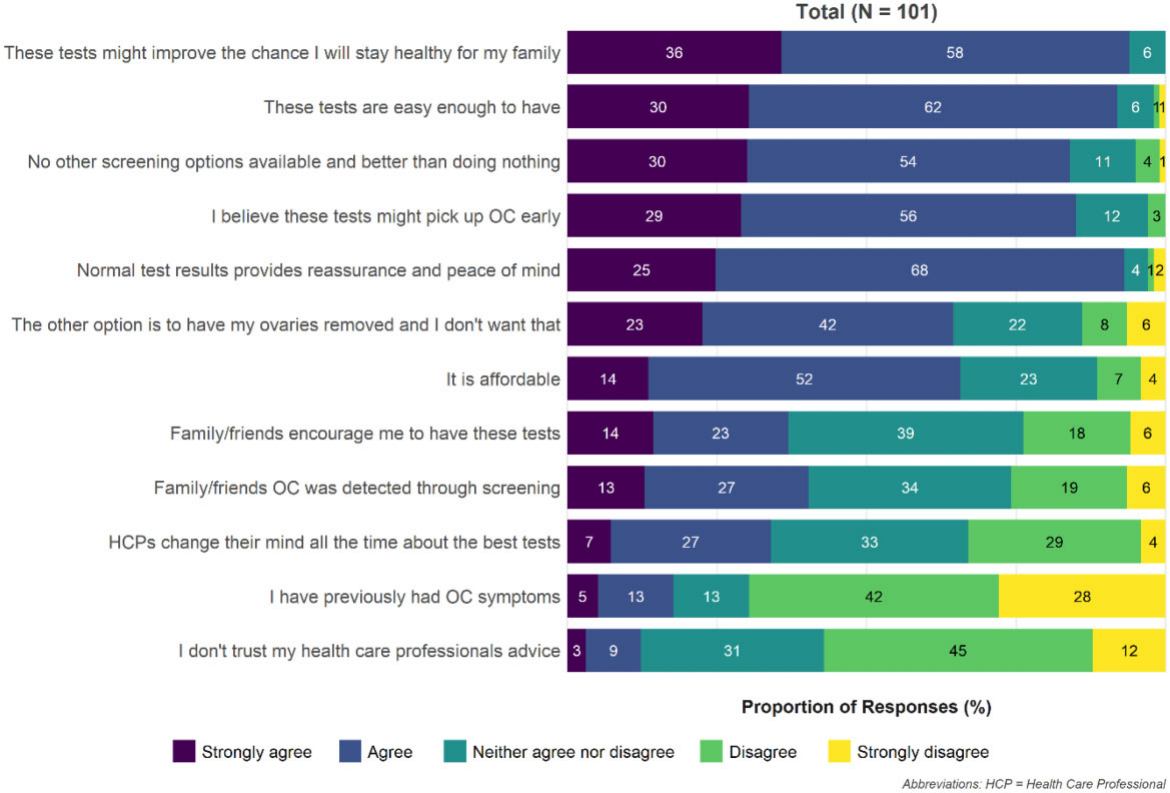
kConFab women: reasons to continue ovarian cancer (OC) screening. HCP = healthcare professionals; kConFab = Kathleen Cuningham Foundation Consortium for Research into Familial Breast Cancer.

Those women who indicated they would continue screening despite being told it is ineffective were asked their level of agreement with 12 potential reasons to continue screening (Figure 2;Supplementary Table 2, available online). The most frequently endorsed reason mapped to the social role and goals domain of the TDF (“these tests might improve the chance I will stay healthy for my family,” 93.9% [35.7% strongly agreed, 58.2% agreed]). This was followed by “normal test results provide reassurance and peace of mind” (93.1% [24.8% strongly agreed, 68.3% agreed]) in the emotion and reinforcement domains and “these tests are easy enough to have” (91.9% [30.3% strongly agreed, 61.6% agreed]) in the beliefs about capabilities domain. Two-thirds of women (65.6%) agreed that affordability of the tests was a reason to continue screening.

### Clinician Survey

Of 399 FPs identified by kConFab women, 10 were excluded (not practicing [n = 5]; invalid address [n = 5]). Of 148 gynecologists identified, 6 were excluded (not practicing [n = 3]; invalid address [n = 3]). Overall, 252 clinicians of 531 (192 FPs, 60 gynecologists) responded to the survey (response rate = 47.4%, FPs = 49.3%, gynecologists = 42.2%). Clinician characteristics are shown in Table 1. There were no statistically significant differences between clinician responders and nonresponders in terms of sex and date of first medical registration (*P* = .06 and .36, respectively).

A minority of clinicians (FPs = 45.8%, gynecologists = 16.7%) thought OC screening was useful. More than one-third of FPs (n = 77, 40.1%) had ordered ultrasound screening, and 92 (47.9%) had ordered CA125 in the past 2 years. Half of gynecologists (n = 30, 50.0%) had ordered ultrasound screening and 25 (41.6%) CA125.

**Figure 3.**
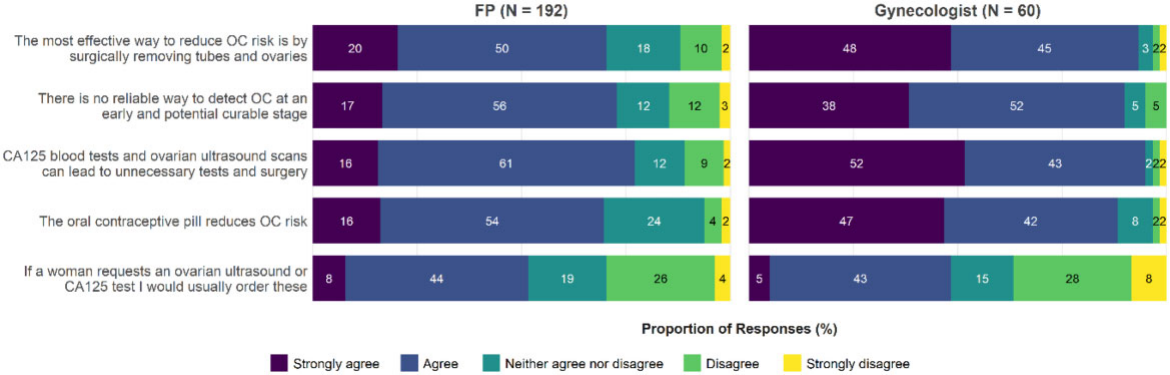
Clinician knowledge about ovarian cancer (OC) screening and risk reduction. CA125 = cancer antigen 125; FP = family physician.

Clinicians were asked to rate their level of agreement with statements about OC screening and risk reduction (Figure 3;Supplementary Table 3, available online). The majority of clinicians agreed there is no reliable way to detect OC at an early stage (72.9% FPs, 90.0% gynecologists) and that CA125 and ultrasound can lead to unnecessary tests and surgery (77.1% FPs, 95.0% gynecologists). However, about half of clinicians (51.6% FPs, 48.3% gynecologists) agreed that they would usually order a CA125 and ultrasound at patient request.

**Figure 4.**
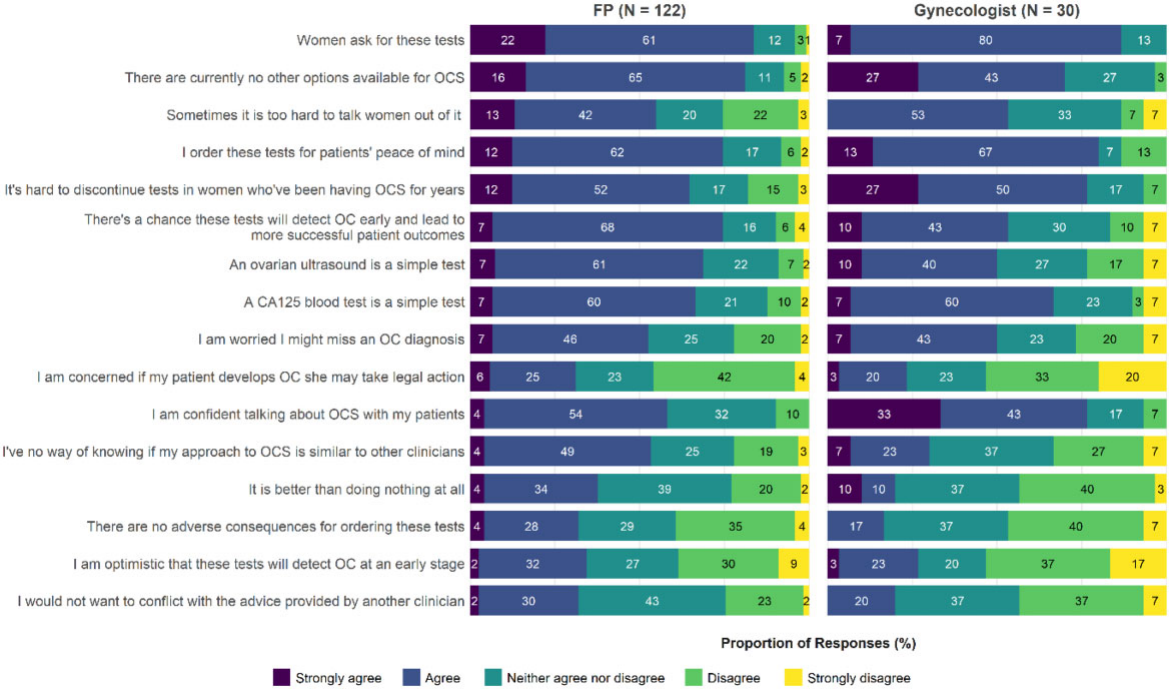
Clinicians: reasons for ordering ovarian cancer screening (OCS). CA125 = cancer antigen 125; FP = family physician.

Clinicians who had ordered OC screening in the past 2 years were asked to identify the strongest motivators for ordering these tests and their level of agreement with each motivator (Figure 4;Supplementary Table 4, available online). The most frequently identified motivators for FPs were “women ask for these tests” (20.7%, TDF social influence domain), the chance these tests will detect OC early and lead to more successful patient outcomes (16.4%, goals domain), for patients’ peace of mind (13.8%, emotion domain), and no other available screening options (11.2%, environmental context and resources domain). For gynecologists, the strongest reasons were no other available screening options (27.6%, TDF environmental context and resource domain), for patients’ peace of mind (17.2%, emotion domain), and difficulty discontinuing tests in women having OC screening (13.8%, emotion domain). Concern about legal action was never the strongest facilitator (0%), but 31.1% of FPs and 23.3% of gynecologists endorsed it as a reason for ordering OC screening.

## Discussion

This Australia-wide study of a large number of women enrolled in a breast cancer cohort, and the clinicians who order their screening, has demonstrated that some women screen for OC despite national guidelines that do not recommend it. Further, we have identified motivators of OC screening, for both women and clinicians, and the behavioral domains in which they operate.

Our study has a number of strengths including a high response rate compared with some others in the literature (6,29) and the administration of surveys developed using an implementation research framework. However, our study had several limitations. The views of nonresponders may differ from those reported: kConFab women who responded were more likely to have a tertiary education, but there were no statistically significant differences between clinician responders and nonresponders for the limited factors measured. It is possible that the screening behaviors of kConFab women differ from the general population, because these long-term cohort study participants may have been exposed to more information about OC screening. We also did not attempt to confirm survey responses using medical record or administrative data.

OC screening does not improve survival and is not recommended by international guidelines. Previous studies have shown that one-third to half of US clinicians order OC screening for average risk women, and those with nonprofessional exposure to OC are more likely to order screening tests (7,30). We deliberately sampled clinicians who identified women as having ordered screening tests for them, so our data do not enable us to generalize about the proportion of clinicians who order OC screening in Australia. Studies have shown that some clinicians consider OC screening to be effective (6,31), and this is consistent with our study, with one-third of clinicians reporting that ultrasound is effective for OC screening. A small proportion of our study sample had objectively increased OC risk. Previous research demonstrated screening uptake between 17% and 31% in high-risk women (29,32). Other studies on beliefs and attitudes of women toward OC screening have focused on average-risk women and excluded women at elevated risk (9,10).

Most clinicians in our study agreed with “there is a chance these tests will detect cancer early and lead to more successful patient outcomes.” This facilitator operates within the goals domain of the TDF and, using the COM-B behavior change wheel (27,33), suggests interventions focused on education, persuasion, and coercion may be effective (Table 1). For example, developing educational resources that explain data from screening trials, illustrating lack of benefit vs harms of screening may prevent misinterpretation of screening efficacy. In addition, communication directed at clinicians by respected experts in each field, outlining the expert’s rationale for not ordering OC screening may be effective in modifying clinician behaviors.

Compared with FPs, the majority (70%) of gynecologists surveyed knew that ultrasound and CA125 testing are not effective for early detection of OC. Despite this, almost half of both FPs and gynecologists had ordered screening in the past 2 years. This demonstrates a discordance of practice among gynecologists: Despite knowledge of ineffectiveness, they continue to order these tests. The strongest motivators for gynecologists (no other options available for OC screening—environmental context and resources) and FPs (women ask for these tests—social influence) map to the intervention functions of restriction, and environmental restructuring in the COM-B behavior wheel. Thus, legislation and regulation of the availability of funded access to OC screening tests are policy categories that should be considered to reduce OC screening. Both clinician groups identified strong motivators within the TDF emotion domain (patient peace of mind and difficulty discontinuing tests). Interventions focused on modeling, enablement, and persuasion are important to modify this automatic motivation. This may be achieved through the type of communication campaign already described above (ie, using a respected leader in the field). Active educational interventions such as workshops on persuasive communication and approaching discontinuing tests with patients may also be effective in equipping clinicians for difficult conversations about ceasing screening.

Patient expectations strongly influence ordering screening (7), and patient request is a strong predictor of nonadherence to OC screening guidelines. In one study, a quarter of clinicians who did not believe OC screening to be effective at least sometimes ordered tests at patient request (5). Thus, although clinician knowledge is important, pressure and expectations from women may continue to result in clinicians ordering OC screening. Targeting the motivators for women to ask for screening tests will be important in reducing OC screening.

kConFab women identified strong motivators for OC screening within the TDF domains of social role (staying healthy for family), emotion (tests provide peace of mind), and beliefs about capabilities (tests are easy enough to have). Lack of knowledge among surveyed women was prominent, suggesting that education illustrating the lack of efficacy and the harms of screening could be central. To target the emotion domain, the COM-B behavioral change theory suggests that persuasion—for example by high-profile women who do not screen because it is potentially harmful—might be useful. Reducing access to screening tests should be considered, especially because 66% of women identified the affordability of tests as a reason to continue screening. This is interesting given that, in Australia, there is no provision for funded ultrasound or CA125 screening. Further research might consider whether women are paying for these tests or accessing them through funded routes (eg, using a symptomatic rather than screening indication) and whether this is in the private or public health setting.

Developing effective interventions to eradicate OC screening in Australia will be challenging. Fear often drives patient behaviors, and removing the sense of control from “doing something” without a replacement intervention will be difficult. Research exploring practical ways to address patient fear in this setting may be beneficial. Passive education, in isolation, rarely results in sustained behavior change and thus needs to be combined with effective system-focused strategies (34). The clinician–patient relationship is complex, and patient expectations can be difficult to modify in this context. A parallel example is the unnecessary prescribing of antibiotics for uncomplicated respiratory tract infections by FPs, which is strongly influenced by patient expectations (35). Research has shown that educational interventions have limited benefit, but system approaches using electronic-based decision support tools and regulatory processes are more effective (36,37). Therefore, implementation of effective interventions to reduce OC screening is likely to require a multifaceted individual and system-based approach addressing the behavioral domains that we have identified as facilitating inappropriate screening (34).

## Funding

This research was supported by Cancer Australia and the National Breast Cancer Foundation (PdCCRS #1100868). kConFab and the kConFab Follow-Up Study have received additional funding support from Cancer Australia (809195), the Australian National Breast Cancer Foundation (IF 17), the Australian National Health and Medical Research Council (454508, 288704, 145684), the National Institute of Health USA (1RO1CA159868), the Queensland Cancer Fund, the Cancer Councils of New South Wales, Victoria, Tasmania and South Australia, and the Cancer Foundation of Western Australia.

KAP is an Australian National Breast Cancer Foundation Fellow (PRAC17-004).

## Footnotes

**Role of the funder:** The funders had no role in the design of the study; the collection, analysis, and interpretation of the data; the writing of the manuscript; and the decision to submit the manuscript for publication.

**Disclosures:** The authors declare no conflicts of interest.

**Disclaimer:** The contents of this manuscript are solely the responsibility of the authors and do not necessarily reflect the views of Cancer Australia or the Australian National Breast Cancer Foundation.

**Acknowledgements:** We thank Sandra Picken, Lucy Stanhope, Sarah O’Connor, Gerda Evans, Leslie Gilham, Heather Thorne, Eveline Niedermayr, Sharon Guo, the kConFab research nurses, and the heads and staff of the Family Cancer Clinics. We thank the women, their families, and the clinicians who participated in this research.

**Author contributions:** All authors of this research paper have directly participated in the planning, execution, and/or analysis of this study. Courtney Macdonald: formal analysis, project administration, visualization, writing – original draft, writing – review and editing. Danielle Mazza: methodology, supervision, validation, writing – review and editing. Martha Hickey: supervision, writing – review and editing. Morgan Hunter: formal analysis, investigation, software. Louise A. Keogh: methodology, supervision, writing – review and editing. kConFab Investigators: data curation, resources, writing – review and editing. Sandra C. Jones: supervision, writing – review and editing. Christobel Saunders: supervision, writing – review and editing. Stephanie Nesci: data curation, funding acquisition, project administration, resources, software. Roger L. Milne: conceptualization, formal analysis, supervision, writing – review and editing. Sue-Anne McLachlan: supervision, writing – review and editing. John L. Hopper: supervision, writing – review and editing. Michael L. Friedlander: supervision, writing – review and editing. Jon Emery: supervision, writing – review and editing. Kelly-Anne Phillips: conceptualization, funding acquisition, formal analysis, investigation, project administration, supervision, writing – original draft, writing – review and editing.

## Data Availability

The data from this study cannot be shared publicly because of ethical and privacy reasons. The data are available on reasonable request to the corresponding author.

## Supplementary Material

pkaa110_Supplementary_Data

## Notes

†These authors contribute equally.
