# Supplementary material for: Motivators of Inappropriate Ovarian Cancer Screening: A Survey of Women and Their Clinicians
Source: JNCI Cancer Spectr. 2020 Dec 8;5(1):pkaa110. doi: 10.1093/jncics/pkaa110 (PMC7853181; doi:10.1093/jncics/pkaa110)
Supplement: pkaa110_Supplementary_Data [file pkaa110_supplementary_data.pdf]

SUPPLEMENTARY MATERIALS

Supplementary Table 1: kConFab women: knowledge about OC screening and risk reduction

Statement	Total (n = 832) (% [95% CI])
**OC screening is recommended for women at an increased risk**	
Strongly agree	216 (26% [23, 29])
Agree	365 (44% [40, 47])
Neither agree nor disagree	73 (9% [7, 11])
Disagree	46 (6% [4, 7])
Strongly disagree	11 (1% [1, 2])
Don't know	121 (15% [12, 17])
**The most effective way to reduce OC risk is by having my ovaries and tubes removed**	
Strongly agree	199 (24% [21, 27])
Agree	210 (25% [22, 28])
Neither agree nor disagree	131 (16% [13, 18])
Disagree	71 (9% [7, 11])
Strongly disagree	25 (3% [2, 4])
Don't know	196 (24% [21, 27])
**I am confident that my doctor will advise me about the best way to manage my OC risk**	
Strongly agree	133 (16% [14, 19])
Agree	374 (45% [42, 48])
Neither agree nor disagree	165 (20% [17, 23])
Disagree	69 (8% [7, 10])
Strongly disagree	24 (3% [2, 4])
Don't know	67 (8% [6, 10])
**There is no reliable way to detect OC early**	
Strongly agree	54 (6% [5, 8])
Agree	180 (22% [19, 25])
Neither agree nor disagree	150 (18% [15, 21])
Disagree	218 (26% [23, 29])
Strongly disagree	41 (5% [4, 7])
Don't know	189 (23% [20, 26])
**Screening for OC can lead to unnecessary tests and surgery**	
Strongly agree	23 (3% [2, 4])
Agree	53 (6% [5, 8])
Neither agree nor disagree	194 (23% [20, 26])
Disagree	265 (32% [29, 35])
Strongly disagree	83 (10% [8, 12])
Don't know	214 (26% [23, 29])
**The oral contraceptive pill reduces OC risk**	
Strongly agree	9 (1% [0, 2])
Agree	63 (8% [6, 10])
Neither agree nor disagree	168 (20% [18, 23])
Disagree	118 (14% [12, 17])
Strongly disagree	28 (3% [2, 5])
Don't know	446 (54% [50, 57])

Supplementary Table 2: kConFab women: reasons to continue OC screening

Motivator	Total (n = 101) (% [95% CI])
**These tests might improve the chance I will stay healthy for my family**	
Strongly agree	35 (36% [26, 46])
Agree	57 (58% [48, 68])
Neither agree nor disagree	6 (6% [2, 13])
Not applicable	3
**These tests are easy enough to have**	
Strongly agree	30 (30% [21, 40])
Agree	61 (62% [51, 71])
Neither agree nor disagree	6 (6% [2, 13])
Disagree	1 (1% [0, 5])
Strongly disagree	1 (1% [0, 5])
Not applicable	2
**No other screening options available and better than doing nothing**	
Strongly agree	30 (30% [21, 40])
Agree	54 (54% [44, 64])
Neither agree nor disagree	11 (11% [6, 19])
Disagree	4 (4% [1, 10])
Strongly disagree	1 (1% [0, 5])
Not applicable	1
**I believe these tests might pick up OC early**	
Strongly agree	29 (29% [20, 39])
Agree	56 (56% [46, 66])
Neither agree nor disagree	12 (12% [6, 20])
Disagree	3 (3% [1, 9])
Not applicable	1
**Normal test results provides reassurance and peace of mind**	
Strongly agree	25 (25% [17, 34])
Agree	69 (68% [58, 77])
Neither agree nor disagree	4 (4% [1, 10])
Disagree	1 (1% [0, 5])
Strongly disagree	2 (2% [0, 7])
**The other option is to have my ovaries removed and I don't want that**	
Strongly agree	21 (23% [15, 32])
Agree	39 (42% [32, 53])
Neither agree nor disagree	20 (22% [14, 31])
Disagree	7 (8% [3, 15])
Strongly disagree	6 (6% [2, 14])
Not applicable	8
**It is affordable**	
Strongly agree	13 (14% [7, 22])
Agree	50 (52% [42, 62])
Neither agree nor disagree	22 (23% [15, 33])
Disagree	7 (7% [3, 14])
Strongly disagree	4 (4% [1, 10])
Not applicable	5
**Family/friends encourage me to have these tests**	
Strongly agree	12 (14% [8, 24])
Agree	19 (23% [14, 33])
Neither agree nor disagree	33 (39% [29, 51])
Disagree	15 (18% [10, 28])
Strongly disagree	5 (6% [2, 13])

Motivator	Total (n = 101) (% [95% CI])
Not applicable	17
**Family/friends OC was detected through screening**	
Strongly agree	8 (13% [6, 24])
Agree	17 (27% [17, 40])
Neither agree nor disagree	21 (34% [22, 47])
Disagree	12 (19% [10, 31])
Strongly disagree	4 (6% [2, 16])
Not applicable	39
**HCPs change their mind all the time about the best tests**	
Strongly agree	7 (7% [3, 14])
Agree	26 (27% [18, 37])
Neither agree nor disagree	32 (33% [24, 43])
Disagree	28 (29% [20, 39])
Strongly disagree	4 (4% [1, 10])
Not applicable	4
**I have previously had OC symptoms**	
Strongly agree	4 (5% [1, 12])
Agree	10 (13% [6, 22])
Neither agree nor disagree	10 (13% [6, 22])
Disagree	33 (42% [31, 53])
Strongly disagree	22 (28% [18, 39])
Not applicable	22
**I don't trust my health care professionals advice**	
Strongly agree	3 (3% [1, 9])
Agree	9 (9% [4, 17])
Neither agree nor disagree	30 (31% [22, 41])
Disagree	44 (45% [35, 55])
Strongly disagree	12 (12% [6, 20])
Not applicable	3

Supplementary Table 3: Clinicians knowledge about OC screening and risk reduction

Statement	Profession		
	FP (n = 192) (% [95% CI])	Gynecologist (n = 60) (% [95% CI])	Total (n = 252) (% [95% CI])
**The most effective way to reduce OC risk is by surgically removing tubes and ovaries**			
Strongly agree	39 (20% [15, 27])	29 (48% [35, 62])	68 (27% [22, 33])
Agree	96 (50% [43, 57])	27 (45% [32, 58])	123 (49% [42, 55])
Neither agree nor disagree	34 (18% [13, 24])	2 (3% [0, 12])	36 (14% [10, 19])
Disagree	19 (10% [6, 15])	1 (2% [0, 9])	20 (8% [5, 12])
Strongly disagree	4 (2% [1, 5])	1 (2% [0, 9])	5 (2% [1, 5])
**There is no reliable way to detect OC at an early and potential curable stage**			
Strongly agree	32 (17% [12, 23])	23 (38% [26, 52])	55 (22% [17, 27])
Agree	108 (56% [49, 63])	31 (52% [38, 65])	139 (55% [49, 61])
Neither agree nor disagree	24 (12% [8, 18])	3 (5% [1, 14])	27 (11% [7, 15])
Disagree	23 (12% [8, 17])	3 (5% [1, 14])	26 (10% [7, 15])
Strongly disagree	5 (3% [1, 6])	0	5 (2% [1, 5])
**CA125 blood tests and ovarian ultrasound scans can lead to unnecessary tests and surgery**			
Strongly agree	30 (16% [11, 22])	31 (52% [38, 65])	61 (24% [19, 30])
Agree	118 (61% [54, 68])	26 (43% [31, 57])	144 (57% [51, 63])
Neither agree nor disagree	23 (12% [8, 17])	1 (2% [0, 9])	24 (10% [6, 14])
Disagree	18 (9% [6, 14])	1 (2% [0, 9])	19 (8% [5, 12])
Strongly disagree	3 (2% [0, 4])	1 (2% [0, 9])	4 (2% [0, 4])
**The oral contraceptive pill reduces OC risk**			
Strongly agree	31 (16% [11, 22])	28 (47% [34, 60])	59 (23% [18, 29])
Agree	103 (54% [46, 61])	25 (42% [29, 55])	128 (51% [44, 57])
Neither agree nor disagree	46 (24% [18, 31])	5 (8% [3, 18])	51 (20% [15, 26])
Disagree	8 (4% [2, 8])	1 (2% [0, 9])	9 (4% [2, 7])
Strongly disagree	4 (2% [1, 5])	1 (2% [0, 9])	5 (2% [1, 5])
**If a woman requests an ovarian ultrasound or CA125 test I would usually order these**			
Strongly agree	15 (8% [4, 13])	3 (5% [1, 14])	18 (7% [4, 11])
Agree	84 (44% [37, 51])	26 (43% [31, 57])	110 (44% [37, 50])
Neither agree nor disagree	36 (19% [13, 25])	9 (15% [7, 27])	45 (18% [13, 23])
Disagree	50 (26% [20, 33])	17 (28% [17, 41])	67 (27% [21, 32])
Strongly disagree	7 (4% [1, 7])	5 (8% [3, 18])	12 (5% [2, 8])

Supplementary Table 4: Clinicians: Reasons for ordering OC screening

Motivators	Profession		
	FP (n = 122) (% [95% CI])	Gynecologist (n = 30) (% [95% CI])	Total (n = 152) (% [95% CI])
**Women ask for these tests**			
Strongly agree	27 (22% [15, 31])	2 (7% [1, 22])	29 (19% [13, 26])
Agree	75 (61% [52, 70])	24 (80% [61, 92])	99 (65% [57, 73])
Neither agree nor disagree	15 (12% [7, 19])	4 (13% [4, 31])	19 (12% [8, 19])
Disagree	4 (3% [1, 8])	0	4 (3% [1, 7])
Strongly disagree	1 (1% [0, 4])	0	1 (1% [0, 4])
**There are currently no other options available for OCS**			
Strongly agree	20 (16% [10, 24])	8 (27% [12, 46])	28 (18% [13, 26])
Agree	79 (65% [56, 73])	13 (43% [25, 63])	92 (61% [52, 68])
Neither agree nor disagree	14 (11% [6, 19])	8 (27% [12, 46])	22 (14% [9, 21])
Disagree	6 (5% [2, 10])	1 (3% [0, 17])	7 (5% [2, 9])
Strongly disagree	3 (2% [1, 7])	0	3 (2% [0, 6])
**Sometimes it is too hard to talk women out of it**			
Strongly agree	16 (13% [8, 20])	0	16 (11% [6, 17])
Agree	51 (42% [33, 51])	16 (53% [34, 72])	67 (44% [36, 52])
Neither agree nor disagree	24 (20% [13, 28])	10 (33% [17, 53])	34 (22% [16, 30])
Disagree	27 (22% [15, 31])	2 (7% [1, 22])	29 (19% [13, 26])
Strongly disagree	4 (3% [1, 8])	2 (7% [1, 22])	6 (4% [1, 8])
**I order these tests for patients' peace of mind**			
Strongly agree	15 (12% [7, 19])	4 (13% [4, 31])	19 (12% [8, 19])
Agree	76 (62% [53, 71])	20 (67% [47, 83])	96 (63% [55, 71])
Neither agree nor disagree	21 (17% [11, 25])	2 (7% [1, 22])	23 (15% [10, 22])
Disagree	7 (6% [2, 11])	4 (13% [4, 31])	11 (7% [4, 13])
Strongly disagree	3 (2% [1, 7])	0	3 (2% [0, 6])
**It's hard to discontinue tests in women who've been having OCS for years**			
Strongly agree	15 (12% [7, 19])	8 (27% [12, 46])	23 (15% [10, 22])
Agree	64 (52% [43, 62])	15 (50% [31, 69])	79 (52% [44, 60])
Neither agree nor disagree	21 (17% [11, 25])	5 (17% [6, 35])	26 (17% [11, 24])
Disagree	18 (15% [9, 22])	2 (7% [1, 22])	20 (13% [8, 20])
Strongly disagree	4 (3% [1, 8])	0	4 (3% [1, 7])
**There's a chance these tests will detect OC early and lead to more successful patient outcomes**			
Strongly agree	8 (7% [3, 13])	3 (10% [2, 27])	11 (7% [4, 13])
Agree	83 (68% [59, 76])	13 (43% [25, 63])	96 (63% [55, 71])
Neither agree nor disagree	19 (16% [10, 23])	9 (30% [15, 49])	28 (18% [13, 26])
Disagree	7 (6% [2, 11])	3 (10% [2, 27])	10 (7% [3, 12])
Strongly disagree	5 (4% [1, 9])	2 (7% [1, 22])	7 (5% [2, 9])
**An ovarian ultrasound is a simple test**			
Strongly agree	9 (7% [3, 14])	3 (10% [2, 27])	12 (8% [4, 13])
Agree	75 (61% [52, 70])	12 (40% [23, 59])	87 (57% [49, 65])
Neither agree nor disagree	27 (22% [15, 31])	8 (27% [12, 46])	35 (23% [17, 31])
Disagree	9 (7% [3, 14])	5 (17% [6, 35])	14 (9% [5, 15])
Strongly disagree	2 (2% [0, 6])	2 (7% [1, 22])	4 (3% [1, 7])
**A CA125 blood test is a simple test**			
Strongly agree	8 (7% [3, 13])	2 (7% [1, 22])	10 (7% [3, 12])
Agree	73 (60% [51, 69])	18 (60% [41, 77])	91 (60% [52, 68])
Neither agree nor disagree	26 (21% [14, 30])	7 (23% [10, 42])	33 (22% [15, 29])
Disagree	12 (10% [5, 17])	1 (3% [0, 17])	13 (9% [5, 14])

Motivators	Profession		
	FP (n = 122) (% [95% CI])	Gynecologist (n = 30) (% [95% CI])	Total (n = 152) (% [95% CI])
Strongly disagree	3 (2% [1, 7])	2 (7% [1, 22])	5 (3% [1, 8])
**I am worried I might miss an OC diagnosis**			
Strongly agree	8 (7% [3, 13])	2 (7% [1, 22])	10 (7% [3, 12])
Agree	56 (46% [37, 55])	13 (43% [25, 63])	69 (45% [37, 54])
Neither agree nor disagree	31 (25% [18, 34])	7 (23% [10, 42])	38 (25% [18, 33])
Disagree	24 (20% [13, 28])	6 (20% [8, 39])	30 (20% [14, 27])
Strongly disagree	3 (2% [1, 7])	2 (7% [1, 22])	5 (3% [1, 8])
**I am concerned if my patient develops OC she may take legal action**			
Strongly agree	7 (6% [2, 11])	1 (3% [0, 17])	8 (5% [2, 10])
Agree	31 (25% [18, 34])	6 (20% [8, 39])	37 (24% [18, 32])
Neither agree nor disagree	28 (23% [16, 31])	7 (23% [10, 42])	35 (23% [17, 31])
Disagree	51 (42% [33, 51])	10 (33% [17, 53])	61 (40% [32, 48])
Strongly disagree	5 (4% [1, 9])	6 (20% [8, 39])	11 (7% [4, 13])
**I am confident talking about OCS with my patients**			
Strongly agree	5 (4% [1, 9])	10 (33% [17, 53])	15 (10% [6, 16])
Agree	66 (54% [45, 63])	13 (43% [25, 63])	79 (52% [44, 60])
Neither agree nor disagree	39 (32% [24, 41])	5 (17% [6, 35])	44 (29% [22, 37])
Disagree	12 (10% [5, 17])	2 (7% [1, 22])	14 (9% [5, 15])
**I've no way of knowing if my approach to OCS is similar to other clinicians**			
Strongly agree	5 (4% [1, 9])	2 (7% [1, 22])	7 (5% [2, 9])
Agree	60 (49% [40, 58])	7 (23% [10, 42])	67 (44% [36, 52])
Neither agree nor disagree	30 (25% [17, 33])	11 (37% [20, 56])	41 (27% [20, 35])
Disagree	23 (19% [12, 27])	8 (27% [12, 46])	31 (20% [14, 28])
Strongly disagree	4 (3% [1, 8])	2 (7% [1, 22])	6 (4% [1, 8])
**It is better than doing nothing at all**			
Strongly agree	5 (4% [1, 9])	3 (10% [2, 27])	8 (5% [2, 10])
Agree	41 (34% [25, 43])	3 (10% [2, 27])	44 (29% [22, 37])
Neither agree nor disagree	48 (39% [31, 49])	11 (37% [20, 56])	59 (39% [31, 47])
Disagree	25 (20% [14, 29])	12 (40% [23, 59])	37 (24% [18, 32])
Strongly disagree	3 (2% [1, 7])	1 (3% [0, 17])	4 (3% [1, 7])
**There are no adverse consequences for ordering these tests**			
Strongly agree	5 (4% [1, 9])	0	5 (3% [1, 8])
Agree	34 (28% [20, 37])	5 (17% [6, 35])	39 (26% [19, 33])
Neither agree nor disagree	35 (29% [21, 38])	11 (37% [20, 56])	46 (30% [23, 38])
Disagree	43 (35% [27, 44])	12 (40% [23, 59])	55 (36% [29, 44])
Strongly disagree	5 (4% [1, 9])	2 (7% [1, 22])	7 (5% [2, 9])
**I am optimistic that these tests will detect OC at an early stage**			
Strongly agree	3 (2% [1, 7])	1 (3% [0, 17])	4 (3% [1, 7])
Agree	39 (32% [24, 41])	7 (23% [10, 42])	46 (30% [23, 38])
Neither agree nor disagree	33 (27% [19, 36])	6 (20% [8, 39])	39 (26% [19, 33])
Disagree	36 (30% [22, 38])	11 (37% [20, 56])	47 (31% [24, 39])
Strongly disagree	11 (9% [5, 16])	5 (17% [6, 35])	16 (11% [6, 17])
**I would not want to conflict with the advice provided by another clinician**			
Strongly agree	3 (2% [1, 7])	0	3 (2% [0, 6])
Agree	36 (30% [22, 38])	6 (20% [8, 39])	42 (28% [21, 35])
Neither agree nor disagree	53 (43% [34, 53])	11 (37% [20, 56])	64 (42% [34, 50])
Disagree	28 (23% [16, 31])	11 (37% [20, 56])	39 (26% [19, 33])
Strongly disagree	2 (2% [0, 6])	2 (7% [1, 22])	4 (3% [1, 7])
